# Scientific approach, attitudes, and perspectives on research among Swedish physiotherapy students—a cross-sectional study

**DOI:** 10.1186/s12909-024-05477-0

**Published:** 2024-05-07

**Authors:** Frida Eek, Kjerstin Stigmar

**Affiliations:** Department of Health Sciences, Lund University, Box 117, Lund, 22100 Sweden

**Keywords:** Physiotherapist education, Scientific knowledge, Scientific attitude, Evidence-based practice

## Abstract

**Background:**

Scientific evidence is important to evidence-based practice. Hence, the application of evidence-based practice requires relevant skills and an understanding of science, which therefore need to be learned and trained during the undergraduate program in physiotherapy. The aim of this study was to investigate attitudes, perceived competence, and conditions for a scientific approach among physiotherapy students in Sweden, and to compare attitudes and perceived competence between students in different program years.

**Methods:**

Physiotherapy students from six universities (*n* = 1499) were invited to respond to a digital survey. The survey contained questions regarding attitudes toward science, perceived competence in research interpretations and open comments regarding requirements for a strengthened scientific approach during education. Comparisons between education years were performed with ANOVA/Kruskal‒Wallis test (scale outcomes) and logistic regression (binary outcomes).

**Results:**

A total of 466 students responded to the survey. In total, 57% (*n* = 266) of the students had a high interest in science. No significant difference in interest in science was found between students in the three program years, but 75% (*n* = 347) reported increased interest during the program. A perceived high ability to understand the structure and performance of scientific studies was reported by 31% (*n* = 144), to evaluate the methodology by 16% (*n* = 72) and to interpret statistical results from scientific studies by 12% (*n* = 55). The lowest perceived competence was reported among students in their second year (*p* < 0.05). A majority of the students (88%; *n* = 410) reported a perceived personal need for strengthened conditions for a scientific approach, with suggested prerequisites during education via increased theoretical and applied understanding of the research.

**Conclusion:**

Even though this study does not fully cover physiotherapy students at all undergraduate programmes in Sweden, the results support that a scientific approach and training should be strengthened during education to enable physiotherapists to understand and interpret science and to fully apply an evidence-based approach in upcoming clinical practice. Both theoretical and applied knowledge and understanding are needed.

**Supplementary Information:**

The online version contains supplementary material available at 10.1186/s12909-024-05477-0.

## Introduction

All clinical practice should be evidence based. Evidence-based practice (EBP) rests on three concepts: scientific evidence, clinical expertise, and patient preferences and context [1, 2]. The scientific evidence is an important part of EBP [3]. As a clinician, it is important to not only be able to apply research in clinical practice but also to be able to value the quality of research and consider whether the results are valid and should be employed in clinical practice. The EBP process contains five steps: (1) “ask”: define a question, (2) “acquire”: search and track down the best evidence with response to the question; (3) “appraise: critically evaluate the evidence”, (4) “apply”: integrate the evidence with clinical expertise and patients’ preferences and individual circumstances; (5) “assess”: evaluating the effectiveness and efficiency of stages 1–4 [3, 4]. Consequently, the EBP process includes and requires the ability to search for, interpret, and critically evaluate scientific results. This requires relevant skills and an understanding of science, which need to be learned and trained during the undergraduate program in physiotherapy. In clinical physiotherapy, the responsibility for the decision on treatment and rehabilitation forms often rests with the individual physiotherapist (PT) and/or close colleagues since a stated consensus or clinical guidelines are not always available. Even though clinical guidelines exist within physiotherapy, they are not always applied [5].

Several studies have investigated PTs’ attitudes toward and perceptions of the application of EBP and have indicated that the EBP process is often not fully applied and that PTs prefer to obtain knowledge from colleagues or social networks rather than through the scientific literature [4]. In Swedish studies, PTs have reported barriers to applying EBP, mainly as lack of time, lack of availability/accessibility to scientific literature, lack of advisers, lack of knowledge and lack of interest among superiors [6, 7]. Additionally, in studies from Colombia and Austria, insufficient knowledge and understanding of research and statistics have been indicated as the main barrier to EBP among PTs [8, 9]. This has also been confirmed in systematic reviews, which include quantitative studies where the obstacles to the application of EBP that were most often mentioned were lack of time, lack of support from the employer, lack of both resources and interest, lack of skills regarding the search and critical appraisal of research findings and inability to understand statistics [10, 11].

In two quantitative studies that examined Swedish PTs´ attitudes toward, knowledge of, and approaches to EBP, approximately 90% of the participants agreed that EBP is necessary in clinical practice [7, 12]. In the study by Bernhardsson et al., applying EPB was considered helpful in decision-making by 83% of participants. A large majority (90%) agreed that they wanted to learn or improve the skills needed to apply EBP in the clinic [12]. In the other study, less than half of the participants had a high perceived ability to understand, evaluate and interpret scientific studies and results [7]. Most participants had a high interest in science, but only 40% considered a scientific approach to be generally applied to a high degree within physiotherapy.

Additionally, in a qualitative interview study of Swedish PTs, the participants expressed that knowledge of scientific methods, including the ability to perform critical/analytical reviews, improved the conditions for applying research results in the clinic [13]. Sufficient knowledge and understanding of science are hence crucial prerequisites for EBP since the ability to assess the quality of scientific evidence and to critically appraise results and methodology is an important foundation for EBP [14].

In international studies, participating PTs have mentioned insufficient education and knowledge of scientific methodology to be among the main perceived barriers to applying EBP in clinical practice [8, 9, 15, 16]. In a study of Saudi Arabian PTs, a vast majority considered an understanding of scientific methodology and design to be important for the clinical practice of physiotherapy, that PTs needed to read scientific papers for regular updates on the state of knowledge, and that scientific methodology should be included in PT education [15]. However, the actual knowledge among participants was limited; only a third (or even less regarding some terminology) considered themselves to know and understand well enough to be able to explain to someone else concepts such as EBP as a term or pursuance, randomized controlled trials (RCTs), systematic reviews, risk of bias, effect size and confidence intervals,. In a Brazilian study, PTs considered that the main barrier to implementing EBP was a lack of access to full-text papers; however, a large number of PTs reported insufficient knowledge and understanding and a lack of experience with this topic as barriers to its implementation [16].

Although most studies have focused on examined and clinically working PTs, some studies have explored the perspectives among physiotherapy students. Insufficient time, lack of formal training and research skills, poor ability to critically appraise literature and understanding statistics have also been defined as barriers to EBP among Indian physiotherapy students [17]. The authors of that study highlight the fact that these deficiencies in the level of knowledge persist, even though education within scientific methodology has increased and been implemented during the final year of the program. Effective education is considered the most powerful tool for overcoming barriers toward EBP. A previous study showed that physiotherapy students experience several barriers to the development of knowledge within EBP and that collaboration with clinics during practice is experienced both as an instructive model and hindrance [18]. A Norwegian study including students in different health disciplines showed that students generally considered EBP relevant but revealed a low understanding of terminology, low confidence in EBP skills, and low use of EBP in clinical situations [19]. Additionally, overall low agreement has been found between self-reported and objectively assessed knowledge of EBP terminology, with considerably lower objective values than perceived knowledge [20].

In 2021, the global association World Physiotherapy provided a PT education framework [21], with the intention: “The goal of physiotherapist education is to ensure the continuing development of physiotherapists who are competent and entitled to practise the profession, without limitation, and in accordance with the definition of physiotherapist practice within their individual countries”. Although there might be differences in clinical practice globally, the World physiotherapy framework includes EBP as one of eight competence domains for physiotherapist practice and it should therefore be applied, regardless of specific country,

Hence, there are strong indications that the prerequisites for the application of a scientific approach should be strengthened during undergraduate education, so knowledge and understanding of scientific methodology can be founded and fortified both for improved intake of knowledge during education and for later application of EBP.

The aim of this study was therefore to investigate attitudes toward science, as well as perceived competence and conditions for a scientific approach among physiotherapy students at the undergraduate program in physiotherapy in Sweden. A further aim was to compare attitudes and perceived competence among physiotherapy students in different years of education.

## Method

### Design and population

A cross-sectional study was conducted via a digital/web-based survey. The study was directed to students at all undergraduate PT programs in Sweden and collected during the spring and autumn semesters of 2022.

### Setting

To become a registered PT in Sweden, examination from a three-year undergraduate program (180 credits) is required. After completed education, the students can apply for a Degree of Bachelor of Science in Physiotherapy, which is a prerequisite for a formal registration as PT from the National Board of Health and Welfare. They can also apply for a Degree of Bachelor of Medical Science with a Major in Physiotherapy. The undergraduate program in physiotherapy is available at eight universities in Sweden. All programs include a bachelor thesis, but the specific program structure is designed by each university All programs must however meet the overarching national graduation goals outlined by the Higher Education Ordinance (Högskoleförordningen SFS 1993:100 appendix 2). Among these goals, several relate to a scientific approach (see box below).

**Table:** 

National graduation goals related to scientific approach: For the Degree of Bachelor of Science in Physiotherapy, the student should:
– Demonstrate knowledge of the scientific foundation of the field and awareness of current research and development work, as well as knowledge of the relationship between science and proven experience and its importance for professional practice – Demonstrate the ability to critically examine, assess, and utilize relevant information, as well as to engage in discussions on new facts, phenomena, and issues with various groups, thereby contributing to the development of the profession and the field – Demonstrate the ability to, with a holistic perspective on the individual, make assessments of interventions based on relevant scientific, societal, and ethical aspects, with particular consideration for human rights
**For the Degree of Bachelor of Medical Science with a Major in Physiotherapy, the students should**: – Demonstrate knowledge and understanding within the main area of the education, including knowledge of the scientific basis, knowledge of applicable methods within the area, acquiring a deeper knowledge within some part of the area, and orientation within current research issues. – Demonstrate the ability to seek, collect, evaluate, and critically interpret relevant information in an issue, as well as to critically discuss phenomena, questions, and situations – Demonstrate the ability to, within the main field of education, make assessments considering relevant scientific, societal, and ethical aspects

### Sample selection and data collection

Program directors at all eight undergraduate physiotherapy programs at universities in Sweden were contacted and asked to distribute the invitation and link to all students at their respective program. The main suggestion was to present the study and invitation to students during a teaching session/lecture with a QR link where the students could connect and respond to the survey directly via mobile phone and to distribute an email with a link to the survey for those who were provided to respond via computer. Six programs agreed to participate, with different approaches. Two programs agreed to perform the main suggested strategy and provided contacts with course-responsible teachers at all (first to sixth) semesters. The invitation and link to the survey were presented during the teaching session (either via the responsible teacher or via the first author (FE)), who was given the opportunity to visit and invite students during a course moment via a digital meeting (zoom). The invitations and links were also distributed via e-mail to all the students. Two programs distributed/published the invitation and link to the survey via a digital learning platform. Two programs did not agree to distribute invitations via the program but referred to student associations, who spread invitations via e-mail and social media channels (one program) or only social media channels (one program).

### Questionnaire

The survey was constructed with the digital survey tool Survey&Report (Artisan Global Media). The content was constructed in collaboration with the authors, based on the three aspects in the aim: attitudes, perceived competence, and conditions for a scientific approach. Questions/items were formulated related to each aspect. The questions related to attitude and perceived competence were similar to those used in a recent study exploring the scientific approach among clinical physiotherapists [7]. The questions regarding conditions were related to the education. The items related to each aspect are presented below. The survey also included questions regarding background information on sex, age, and current semester at the undergraduate program in physiotherapy. The survey was not tested for reliability or validity. However, it was pilot tested by six students who were asked to give feedback on the relevance and understanding/interpretability of the items. We received only minor comments, and therefore no major further changes were made.

### Attitudes to science and scientific approach

The survey included questions about the student’s level of interest in science, if/how the interest had changed during education, how important a scientific approach was considered to be for quality of clinical practice, and to what degree the general clinical practice within physiotherapy in Sweden was considered to be based on scientific evidence.

The response alternatives were five categorical Likert scales (the response alternatives for each question are presented in Table 1). An open-ended question was asked regarding the reason for the changed interest in research during education.

**Table 1:** Sample description

	Total *n* = 466	Year 1 *n* = 139	Year 2 *n* = 164	Year 3 *n* = 163
**Sex% (n)** Total *n* 465				
Female	61.4 (286)	60.4 (84)	65.9 (108)	57.7 (94)
Male	37.8 (176)	39.6 (55)	34.1 (56)	39.9 (65)
Don’t want to declare	0.6 (3)	0	0	1.8 (3)
**Age** Mean (sd) years Total *n* = 457	26.5 (6.5)	25.1 (6.4)	27.2 (7.1)	27.1 (5.9)
Md (Q1-Q3)	24 (22–29)	23 (21–28)	24 (22–30)	25 (23–30)
**University/recruitment % (n)** Total *n* = 466				
University A/invitation during teaching session	35.2 (164)	30.2 (42)	50.0 (82)	24.5 (40)
University B/invitation during teaching session	32.2 (150)	38.1 (53)	28.0 (46)	31.3 (51)
Remaining universities/invitation via email/Social media	32.6 (152)	31.7 (44)	22.0 (36)	44.2 (72)

### Uptake and perceived competence to assimilate science

Participants were asked how often they read scientific articles about health, medicine, or physiotherapy (regularly/often, sometimes, rarely, or never) and how many articles they usually read during a month. If they stated that they had read scientific papers only sometimes or rarely or never, they were asked which suggested reasons they agreed with for not reading scientific papers. Questions regarding their perceived ability to understand the structure and performance of scientific studies, evaluate methodology/performance, and interpret statistical results were asked with response alternatives on a five-point Likert scale (very low (1), very high (5), or not know/can’t judge).

### Conditions for a scientific approach

The participants were further asked to what extent they perceived that a scientific approach had been applied within the undergraduate program in physiotherapy (not at all, unclear or sporadically, clearly, and consistent, or very clearly and consistent) and to what extent teaching within scientific methodology had been present during the education (sufficient, too little, or too much) and if they considered a personal need for strengthened scientific approach. Via an open-ended question, the participants were further asked what they thought would be needed to strengthen their scientific approach and ability.

### Data management and statistics

Most five categorical Likert scales were dichotomized (into two categories such as, e.g., “quite/very” vs. “moderately/little/not at all” interested) and presented as percentages (%) and numbers (n) for each category in the total sample and for each program year (1^st,^ 2nd, or 3rd ). Categorical outcomes were compared between program years with the chi-square test, and if the difference was statistically significant, the binary outcomes were pairwise compared with the z test. The odds for binary outcomes were further compared between students in different program years via logistic regression and presented with odds ratios with corresponding confidence intervals. Since the accepted/applied invitation strategies differed between the programs, the universities were categorized into three categories: two categories, with students from each of the two universities actively collecting the data during teaching (University A and University B); and the third category (“remaining universities”), with students from universities where recruitment/invitations were performed via email/social media/course platforms. To check whether potential differences between programs and/or among the different selection strategies resulted in selection/nonresponse bias that affected the comparisons between years, this variable was included in the logistic regression. However, there were no significant differences between the categories, and inclusion of the variable did not affect the estimated odds ratios; therefore, the selection variable was not included in the final and presented analyses.

The perceived ability to understand and evaluate aspects of science was also presented as the mean score of the scale from 1 (very low) to 5 (very high) and was compared with analysis of variance (ANOVA). If the omnibus test was statistically significant, a post hoc pairwise comparison (LSD) was performed. The number of read papers/month is presented as both the mean and median, but due to the skewed distribution, comparisons between program years were performed with the Kruskal–Wallis test, with post hoc pairwise comparisons.

For open-ended questions regarding perceived reasons for increased or decreased interest in science and what is considered needed for strengthening scientific approach and ability,

The comments were read by the two authors and sorted based on similarities in content into sub- and main categories. For comments that included different perspectives, each perspective was sorted separately. The categories and number of comments within each category are presented in the tables.

### Ethics

All data were collected anonymously. The participants were informed about the study aim and data handling and gave informed consent before access to the survey. Since no intervention was performed on a research subject and no sensitive or personal data/information was handled, the study was not covered by Swedish Ethical Review act (SFS 2003: 460 https://etikprovningsmyndigheten.se/en/what-the-act-says/). However, an application was sent to the Swedish Ethical Review Authority (Dnr 2022-00815-01), who waived the need for ethical approval and confirmed that the study was not covered under Swedish Ethical Review act, and instead provided an advisory opinion with no contradictions to the performance of the study.

## Results

**Fig. 1:**
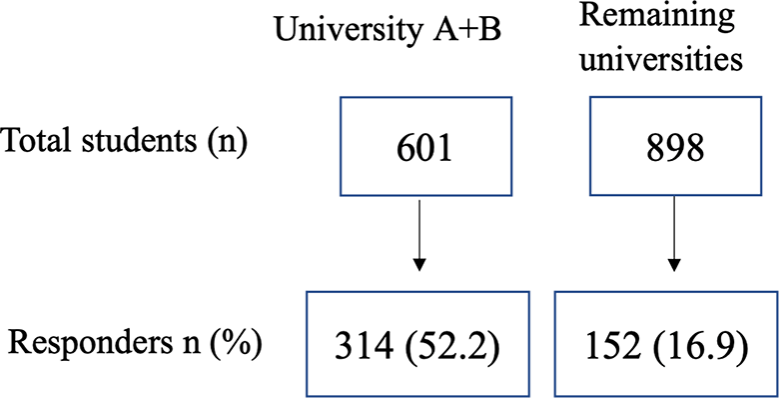
Invitations and response rates from students at universities with different invitation/collection strategies. University A + B: invitation/data collection actively during teaching moment Remaining universities: invitations via email/social media/course platform

In total, 466 students responded to the survey (Fig. 1), of which 61% female (*n* = 276) and 38% male (*n* = 176), with mean age 26.5 (SD 6.5) (Table 2).

**Table 2:** Interest in and perceptions of science and evidence-based practice among students at the undergraduate programs in physiotherapy

	Total *n* = 466	Year 1 *n* = 139	Year 2 *n* = 164	Year 3 *n* = 163	*p*-value*
Interest in science					
Quite/very interested % (n)	57.1 (266)	55.4 (77)	58.5 (96)	57.1 (93)	
Moderate/Little/not interested % (n)	42.9 (200)	44.6 (62)	41.5 (68)	42.9 (70)	0.859
Quite/very interested Odds ratio (95% CI)	–	1 (ref)	1.14 (0.72–1.79)	1.07 (0.68–1.69)	
**Change in interest during education**					
Increased % (n)	74.5 (347)	61.9 (86)	79.9 (131)^a^	79.8 (130)^a^	
No change/decrease % (n)	25.5 (119)	38.1 (53)	20.1 (33)	20.2 (33)	< 0.001
Increased interest Odds ratio (95% CI)	–	1 (ref)	**2.45 (1.47–4.09)**	**2.43 (1.45–4.06)**	
**Importance of scientific approach in the clinical practice of physiotherapy**					
High/very high importance % (n)	91.0 (424)	94.2 (131)^c^	93.9 (154)^c^	85.3 (139)^ab^	
Moderate/low/none% (n)	9.0 (42)	5.8 (8)	6.1 (10)	14.7 (24)	0.007
High/very high importance Odds ratio (95% CI)	–	1 (ref)	0.94 (0.36–2.45)	**0.35 (0.15–0.82)**	
**Perception of to what degree clinical practice among Swedish PTs generally is based on scientific evidence/basis ** **%** (n)					
High/very high degree	52.4 (244)	62.6 (87)^c^	54.9 (90)^c^	41.1 (67)^ab^	
Moderate/low/not at all/don’t know	47.6 (222)	37.4 (52)	45.1 (74)	58.9 (96)	< 0.001
High/very high degree Odds ratio (95% CI)	–	1 (ref)	0.73 (0.46–1.15)	**0.42 (0.26–0.66)**	

Bold numbers: significant (*p* < 0.05) odds ratio

*Chi square test

^a b c^ Significant (*p* < 0.05) pairwise difference from a = 1st year b = 2nd year, c = 3rd year

### Attitudes to science and scientific approach

The students’ interest in science was fairly high, and their interest did not differ significantly between program years. However, most students considered themselves to have gained increased interest during the undergraduate program, to a greater extent among students during program years 2 and 3 (Table 1). The majority of the students considered a scientific approach to be highly important for clinical practice, but the odds of this approach were significantly lower among final-year students (Table 1). However, only half of the students believed that clinical physiotherapy practice in Sweden in general is based mainly on scientific evidence, with the lowest odds among students in their final year.

The main reasons for increased interest in science, reported via open comments, were increased knowledge and understanding of science, more exposure to research and more inspiring lectures. The students also reported more insight into the importance of science, both regarding clinical practice and the ability to evaluate and argue for relevant development within the profession, as a reason for increased interest. Only a few students reported decreased interest in science because they found too much attention on research during the undergraduate program and because science was experienced as boring and difficult (Table 3).

**Table 3:** Reasons for changed interest in science (categorized open comments)

Increased interest		Decreased interest
Education has increased interest (*n* = 216)	Increased insight in science and its importance (*n* = 137)	The undergraduate program in physiotherapy has rendered in less interest in research (*n* = 9)
-Increased knowledge and understanding (*n* = 97) -More exposed for research and better access to research (*n* = 69) -Inspiring lectures with interesting topics (*n* = 50) -The work with the bachelor´s thesis (*n* = 7)	-Insight in the necessity for own clinical practice (*n* = 51) -General importance to be updated and able to argue for evidence-based medicine (*n* = 83) -Wants to engage in research (*n* = 3)	-Too much focus on science instead of practice -Unmotivating/boring /difficult

### Uptake and perceived competence to assimilate science

A very small percentage (7.9–11.6%) of students read scientific articles regularly or often during the first two years of the program (Table 4). On average, among the first-year students, 1.8 (SD 2.0) articles were read/month, and during the second-year program, 2.5 (SD 2.8)/month, with a median of 1 (IQR 1–2) and 2 (IQR 1–3) were read/month. The proportion of students who read often/regularly was significantly greater at year 3, but a considerable number of students still read only occasionally or rarely (Table 4).

**Table 4:** Habit of reading scientific papers, perceived ability to understand and interpret methodological aspects and perspectives on scientific approach during education program

	Total *n* = 466	Year 1 *n* = 139	Year 2 *n* = 164	Year 3 *n* = 163	*p*-value*
**Frequency of reading scientific articles about health, medicine, or physiotherapy % (n)**					
Regularly/often	16.3 (76)	7.9 (11)	11.6 (19)	28.2 (46)	
Sometimes	43.8 (204)	30.2 (42)	47.6 (78)	51.5 (84)	
Rarely/never	39.9 (186)	61.9 (86)	40.9 (67)	20.2 (33)	< 0.001
Read regularly/often; Odds ratio (95% CI)	–	1 (ref)	1.53 (0.70–3.33)	**4.58 (2.26–9.25)**	
**Number of scientific articles read during an ordinary month**					
Mean (SD)	3.1 (3.8)	1.8 (2.0)	2.5 (2.8)	4.7 (5.1)	
Median (IQR)	2 (1–4)	1 (1–2)^bc^	2 (1–3)^ac^	3 (2–5)^ab^	< 0.001
**Perceived ability to understand the structure and performance of scientific studies (study design, etc.)**					
High/very high % (n)	30.9 (144)	28.1 (39)^c^	25.6 (42)^c^	39.1 (63)^ab^	0.02
Odds ratio (95% CI)	–	1 (ref)	0.88 (0.53–1.47)	**1.65 (1.01–2.68)**	
Mean (SD) score 1 (very low) -5 (very high)	3.2 (0.7)	3.2 (0.8)^c^	3.1 (0.7)^c^	3.4 (0.7)^ab^	< 0.001
**Perceived ability to evaluate the methodology of scientific studies (identify bias, assess quality, etc.)**					
High/very high % (n)	15.5 (72)	15.8 (22)^b^	7.9 (13)^ac^	22.8 (37)^b^	< 0.001
Odds ratio (95% CI)	–	1 (ref)	**0.46 (0.22–0.95)**	1.57 (0.88–2.83)	
Mean (SD) score 1 (very low) -5 (very high)	2.9 (0.7)	2.9 (0.8)^b^	2.7 (0.7)^ac^	3.0 (0.7)^b^	< 0.001
**Perceived ability to interpret statistical results from scientific studies (significance/p-value, effect sizes, risk measures, confidence intervals, etc.)**					
High/very high % (n)	11.8 (55)	13.7 (19)	9.8 (16)	12.3 (20)	0.558
High/very high Odds ratio (95% CI)	–	1 (ref)	0.68 (0.34–1.39)	0.89 (0.45–1.74)	
Mean (SD) score 1 (very low) -5 (very high)	2.6 (0.9)	2.6 (0.9)^b^	2.4 (0.9)^ac^	2.7 (0.8)^b^	0.006
**Perception of how the scientific approach has been emphasized throughout the education**					
Not at all/unclear/sporadic % (n)	14.6 (68)	12.2 (17)	14.0 (23)	17.3 (28)	
Clear/very clear and constant % (n)	85.2 (397)	87.8 (122)	86.0 (141)	82.7 (134)	0.449
Clear/very clear and constant Odds ratio (95% CI)	–	1 (ref)	0.85 (0.44–1.67)	0.67 (0.35–1.28)	
**Perception of scope for scientific methodology within the education program**					
Sufficient/enough % (n)	65.9 (307)	79.1 (110)	57.3 (94)	63.2 (103)	
Too little % (n)	29.4 (137)	17.3 (24)	40.9 (67)	28.2 (46)	
Too much % (n)	4.7 (22)	3.6 (5)	1.8 (3)	8.6 (14)	< 0.001
**Perceived personal need for strengthened scientific approach/knowledge**					
Yes % (n)	88.4 (410)	89.2 (124)^bc^	95.7 (155)^ac^	80.4 (131)^ab^	< 0.001
Odds ratio (95% CI)	–	1 (ref)	**2.68 (1.06–6.77)**	**0.50 (0.26–0.96)**	

**Bold numbers**: significant (*p* < 0.05) odds ratio

*Comparison of Categorical outcomes: Chi^2^ test, Mean values: ANOVA, Rank order (median presentation): Kruskal Wallis

^a b c^ Significant (*p* < 0.05) pairwise difference from a = 1st year b = 2nd year, c = 3rd year

**Fig. 2:**
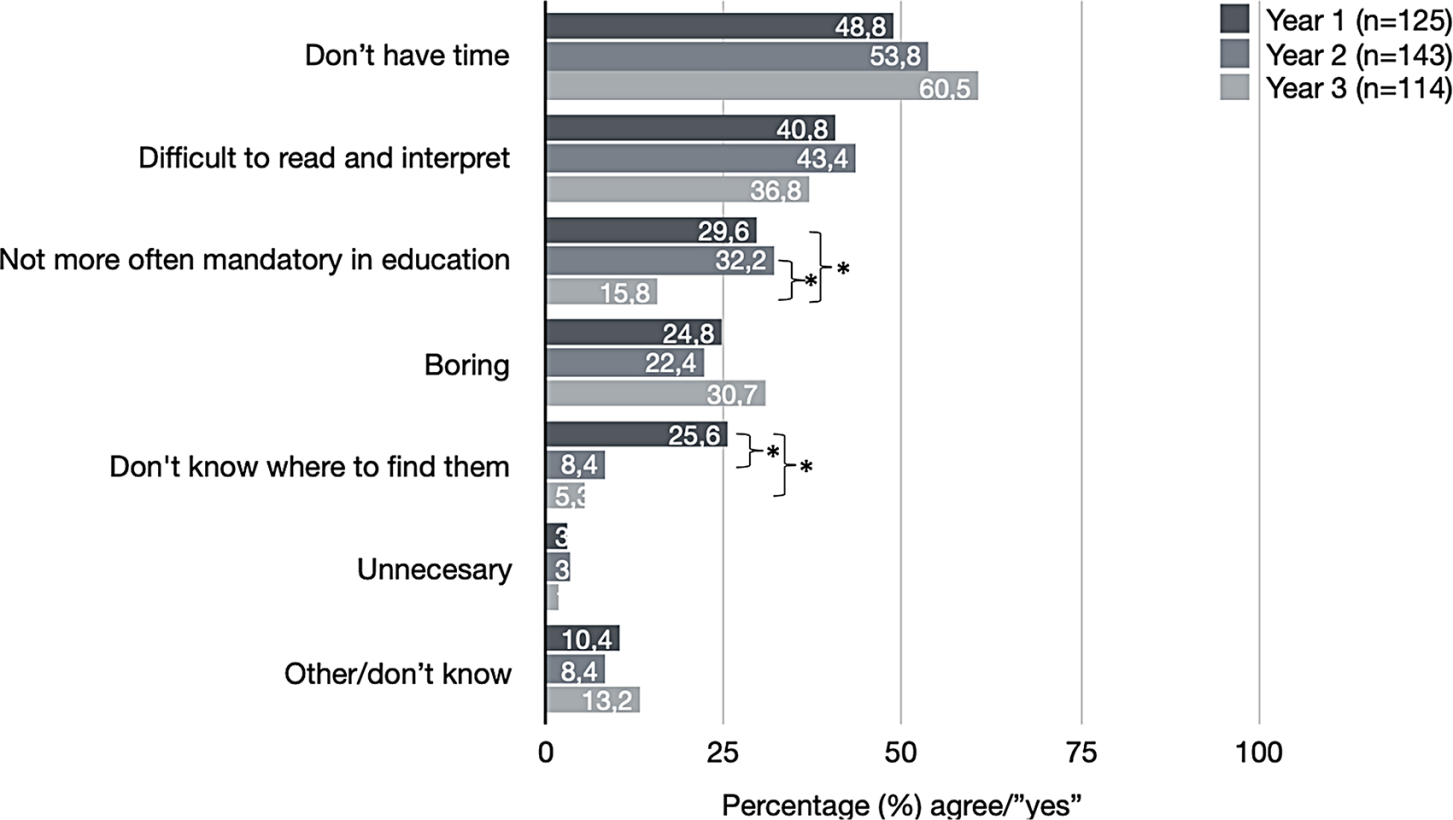
Reasons for not reading scientific paper more often/regularly. *Significant differences (*p* < 0.05)

Among the students who did not read scientific papers regularly, the main reasons were lack of time, difficulty reading and interpreting, and not being more mandatorily included in the education. It was also considered boring, and during the first year, knowledge about where/how to find relevant papers was limited (Fig. 2).

Approximately one-third of the students considered themselves to have a high ability to interpret the structure of studies, with a significantly greater perceived ability among students in program year 3 (Table 4). The proportion of students who considered themselves to have a high ability to value the quality of performance/methodology and interpret statistics was lower. The perceived ability to both value the implementation of the studies and interpret statistics was lowest among students in the second program year; these values were significantly lower than those of both first- and third-year students (Table 4).

### Conditions for a scientific approach

Most students considered that teaching about scientific methodology had a fair amount of space during education, but approximately one-third of final-year students still considered that teaching within this field had too little space. With a similar proportion within all academic years, most of the students considered the scientific approach to be clearly emphasized during education. However, more than half of the students in all three program years considered that their own scientific approach needed to be strengthened, although a significantly lower proportion of final-year students (Table 4). The open question about what is needed to strengthen their scientific approach (Table 5) showed that to strengthen their scientific approach and ability, the students highlighted the need for more knowledge and understanding of scientific methodology, mainly understanding an interpretation of statistics and ability for quality evaluation of scientific papers. More education was requested, with teaching not only spread out during the undergraduate program but also as separate courses. The need for early introduction during the program was also highlighted. The continuous application of acquired and developing knowledge by reading and discussing the content and design was also emphasized as a main requirement. Teachers could also make more efforts to make science relevant and interesting by applying relevant examples from clinical practice and research. Some students reported that their scientific approach and ability is expected to increase during further education, such as master’s studies, and experience in clinical practice (Table 5).

**Table 5:** Perception of what is needed to strengthen your scientific approach and ability (categorized open comments)

Increased knowledge and understanding (*n* = 144)	Increased training and application (*n* = 154)	Effort to increase interest and make science more relevant to the students (*n* = 40)	Approach/ability expected to be achieved with more experience (*n* = 34)
-More knowledge about methodology (*n* = 67) -More teaching (and earlier introduction) (*n* = 66) -More knowledge in searching for evidence (*n* = 11)	-Training in reading and interpretation (*n* = 118) -More allocated time (*n* = 36)		-Further education is expected to increase scientific approach (*n* = 25). -Clinical practice will allow application (*n* = 9).

## Discussion

In this study, we found that a majority of students reported increased interest in science during the undergraduate program in physiotherapy. The main considered reason for the increased interest was developed knowledge and understanding of research and the importance of science. We however found only very small, and no statistically significant, differences in interest between students in the three different program years. Having a scientific approach was considered important, but only approximately half of the students believed that PT practice in general in Sweden was based mainly on science. Most students reported limited ability to understand and interpret scientific methodology and results. Although a scientific approach had been emphasized during the program, the students reported and described a perceived personal need for strengthened conditions for a scientific approach.

In total, about one third of the students (at the six universities that accepted to participate in the study), responded to the survey. Students who participate could be expected to have a greater interest in and more positive attitude toward science than non-responders, but almost half of the respondents still reported moderate or low interest. However, the majority found it important in clinical practice. This is in line with how clinically working PTs reported in a recent study [7]. But while most of the students in our current study considered a scientific approach to be important in clinical practice, only 50% thought that clinical practice was based on science to a high extent, with a lower share (40%) among third year students. In the recent study, 70% of the working PTs considered that their own clinical practice was based on science toa high degree, but they also had a more critical perspective on the general application in clinical practice, where only 40% considered it to be generally based on science to a high degree [7] .

Despite a generally positive attitude toward science, the perceived knowledge and understanding of methodological aspects were limited, especially regarding the evaluation of methodological quality and the understanding and interpretation of statistics. This limitation has been shown in previous studies, both regarding students [17, 18] and clinically active PTs [4, 7, 10, 15, 17, 18]. Some of these studies mirror the situation from some years ago. Since there is a steadily growing body of science within the field of physiotherapy, there are reasons to believe that this could positively impact upon the undergraduate programs. However, both the current study and the study regarding scientific approach among working PTs [7] still show similar aspects as previous studies, which indicates that the conditions for a scientific approach during the undergraduate program still needs to be reinforced.

Additionally, since an overall low agreement has previously been found between healthcare students’ self-reported and objectively assessed knowledge of EBP terminology [20], the actual knowledge may be even lower than how the students have reported their perceived knowledge. The rated perceived knowledge and understanding were relatively greater for the ability to understand the structure and performance of the study, lower for the ability to evaluate the methodology, and lowest for the interpretation of statistics. A development to improve these lower-rated skills was also considered necessary in order to strengthen the scientific approach.

Previous studies that have investigated students’ perceived skills and views on relevance, terminology, confidence, practice, and support for EBP also revealed relatively low knowledge of the scientific aspects [17, 20]. However, a greater knowledge of terminology has been found among physiotherapy students than among other health science students in Norway [19] and Australia [22]. A potential explanation for this difference was that the undergraduate physiotherapy program has a greater focus on research and methodology, since PTs often use tests based on quantitative studies for diagnosis and treatment. Additionally, the development of knowledge and perspective has been shown to progress throughout the program, with higher levels among students who were later in the undergraduate program [23]. More exposure/training showed associations with higher levels of knowledge and perspectives on relevance. Exposure to EBP in clinical practice during the undergraduate program has been shown to be weakly or moderately associated with self-reported EBP behaviour, abilities, and barriers among physiotherapy students during their final year of education [24]. However, no statistically significant association with the students’ use of scientific evidence in clinical settings was found. This also indicates that the undergraduate education needs to strengthen the ability to apply EBP in clinical settings. Confidence and knowledge about research methodology, statistics, and EBP are shown to increase after completing a course (post graduate), which shows the value of having a course on the subject [25]. This finding is in line with the perspectives of the students in our study regarding reasons for both increased interest in science and requirements for a strengthened scientific approach, where the students mainly lifted the importance of developed knowledge and understanding of science.

The students noted not only the need for courses with a defined focus on scientific methodology but also the need for continuous application and translation of acquired knowledge throughout the program, with relevant relation linked to the current clinical topic.

They requested more of reading, critically reviewing, and discussing scientific articles. Especially during the first two years, the consumption of scientific papers was generally quite low, with some outliers (a few students with very high consumption) affecting the mean and SD. The bachelor thesis, which is usually written during third year, may affect the increased intake of scientific papers during the last year. While lack of time was the most reported reason for not reading scientific papers regularly or often, difficulties to read and interpret was also considered a reason for a large share of the students. Additionally, the fact that the reading of scientific papers was not more often mandatory in the undergraduate program was considered a reason to not read more often/regularly. This shows the importance of providing conditions and teaching for increased knowledge and assimilation within the subject of science.

Even if a continuous update and further development/training of knowledge during upcoming clinical practice are needed for optimal clinical application, the undergraduate program should provide a strong foundation for the conditions for taking in and interpreting research results and hence the scientific approach. As a scoping review concluded, students’ knowledge and understanding of scientific methodology are fundamental requirements for the ability to read and interpret scientific articles, which is also an important skill for clinical education [26]. The studies included in the scoping review highlight the importance of including scientific perspectives in undergraduate programs and developing students’ skills and competence. The value of relating scientific aspects to clinical practice is also highlighted. This is also in line with the students’ reported perspectives in our study, where they in open comments highlight that teachers could make more efforts to make science relevant and interesting by applying relevant examples from clinical practice and research. Also, inspiring lectures with interesting topics was perceived as a reason for increased interest in science during the program.

Learning activities of scientific methodology are hence an important part in the undergraduate program and are required to achieve several of the national examination goals. Also in the recent developed global physiotherapy education framework, the importance of EBP is highlighted [21] and this includes knowledge and skills to be able to digest research. In our study, the reported competence was limited, and the majority of students also reported a perceived personal need for strengthened scientific approach/knowledge. The learning activities can, and preferably should, be included and applied in different forms. The students in our study reported both more concreate teaching for increased knowledge and understanding, and a continuous practical training and application throughout the program, as perceived needs for a strengthened scientific approach. The interest and attitude toward science have been shown to increase during methodology courses [27]. The students’ involvement and engagement is however an important aspect of teaching [28]. Multiple and varied forms of interaction between teachers and students increases the students’ engagement [29]. Active engagement and application from the student is also considered to reduce the gap between students with different conditions and attitudes toward the current subject and learning [30]. Introducing teaching of research methodology and to encourage critical thinking is an important part of the medical curriculum, and may increase the capacity for a scientific approach in the upcoming clinical work [29]. The combination of different forms of teaching, such as methods including lectures, computer sessions, small group discussions, journal clubs, and assignments, is seen as beneficial within evidence-based teaching, compared with a single education intervention [31]. Previous studies have also recommended that the knowledge should be applied in some form of active/practical context [32–34], which is also in line with what the students in our study highlighted in open comments regarding what they considered needed to strengthen their scientific approach and ability. This will hence likely increase the interest and motivation among the students. Also in examinations within clinical subject areas, it is recommended to base it on a ‘scenario’ rather than a purely theoretical question [35]. A systematic review showed inconclusive evidence regarding the best time for EBP introduction during a medical undergraduate education [31]. However, to include research already during first-year student education has been suggested [34], even though the initial introduction does not require a formal full methodology course. Therefore, the combination of formal teaching via methodology course(s), and an introduction and a continuous active application related to the clinical topics throughout the undergraduate program are likely to give the most beneficial structure. This approach will, as the students in our study reported, increase interest in and perceived relevance of science and both improve assimilation during education and facilitate the implication of EBP.

## Method/limitation

The survey was developed specifically for the current study and has not been tested for validity or reliability. To our knowledge, there are no such validated and reliable questionnaires available for those students’ perspectives that we aimed to explore. We see no reason to believe that the survey did not capture the students´ perceptions, and the aim was mainly to investigate the attitudes and perspectives related to the defined items. In the pilot test, we received no feedback regarding difficulty to understand or to respond to the questions. The part of the survey that was related to attitudes and perceived competence was also included in a survey with the aim of examining the scientific approach and attitudes among clinically working PTs [7]. As previously mentioned, the perceived knowledge may not correspond with actual competence [20]. The report, however, still provides a view of perceptions and attitudes.

The response rate was limited, especially at universities where the invitation was distributed only via e-mail, course platforms, and/or social media. However, we were not able to apply the same strategy for data collection at all the included universities, since the final decision was up to each university. This resulted in an uneven distribution of response rates. After checking for this, we found no apparent bias effect on the comparison between years. However, there might still be selection/nonresponse bias, especially at “remaining” universities, since interest in and positive attitudes toward science are likely greater among students who decided to participate in this study, than among non-responders. The level of interest and attitude may hence not be fully representative of the whole Swedish physiotherapy student population. Although the comparisons between years seemed less affected, we could not, based on the cross-sectional design, document actual changes within individuals during the years of education.

## Conclusion

Even though this study does not fully cover all students at all undergraduate programs in physiotherapy in Sweden, the results support that a scientific approach and training should be strengthened during education to enable PTs to understand and interpret science and to fully apply an evidence-based approach when in clinical practice. Both theoretical and applied knowledge and understanding are needed.

### Electronic supplementary material

Below is the link to the electronic supplementary material.

Supplementary Material 1

## Data Availability

The dataset analysed during the current study is available from the corresponding author on reasonable request.

## Abbreviations

CI: Confidence interval
EBP: Evidence-based practice
IQR: Interquartile range (quartile 1; quartile 3)
PT: Physiotherapist
SD: standard deviation
