# Supplementary material for: Scientific approach, attitudes, and perspectives on research among Swedish physiotherapy students—a cross-sectional study
Source: BMC Med Educ. 2024 May 7;24:505. doi: 10.1186/s12909-024-05477-0 (PMC11077752; doi:10.1186/s12909-024-05477-0)
Supplement: Supplementary file 1 — Supplementary Material 1 [file 12909_2024_5477_MOESM1_ESM.pdf]

Translated survey items (only Swedish version was applied):

What semester are you studying now?
S1
S2
S3
S4
S5
S6

How interested are you in research/science?
Very interested
Quite interested
Moderately interested
Little interested
Completely uninterested

Has your interest in science changed during your education?
Yes it has increased a lot
Yes it has increased a little
No, it is unchanged
Yes it has decreased a little
Yes it has decreased a lot

(Filter: if any change (increase or decrease))
What is the reason your interest has changed? (open comment)

What importance do you think a scientific approach has in the clinical practice of physiotherapy?
Very high importance
High importance
Moderate importance
Low importance
No importance

To what degree do you think that the clinical practice of physiotherapists (in Sweden) is generally based on a scientific basis?
To a very high degree
To a high degree
To a moderate degree
To a low degree
To a very low degree
Don’t know/can’t judge

How often do you read scientific articles within health, medicine or physiotherapy?
Regularly/often
Sometimes
Rarely
Never

Approximately how many scientific articles do you read during an ordinary month?

(Filter: If response of reading was sometimes/rarely/never) **What is the reason you don't read scientific articles more often?**
(multiple answer options can be chosen, but indicate the main reason(s))
I don't know how/where to find them
I have difficulty reading and interpreting the content
I think it's boring
I think it feels unnecessary
That it is not more often included as a mandatory part of the education
I do not have time
Other/don’t know
 If other: what is the reason?

How is your ability to understand the structure and performance of scientific studies (study design, etc.)?
Very high
High
Moderate
Low
Very low
Don’t know/cannot judge

How is your ability to evaluate the methodology/performance of scientific studies (identify bias, assess quality, etc.)?
Very high
High
Moderate
Low
Very low
Don’t know/cannot judge

How is your ability to interpret statistical results from scientific studies? (significance/p-value, effect sizes, risk measures, confidence intervals, etc.)
Very high
High
Moderate
Low
Very low
Don’t know/cannot judge

Do you take in research news/scientific information via any of the channels below?
(response alternatvies: never/rarely sometimes/moderately often/much)
Popular science journals
Blogs
Pods
Twitter
Instagram/facebook (groups, specific accounts etc)

How do you consider that the scientific approach has been emphasized through the education?
Very clear and thorough
Clear and thorough
Unclear and/or sporadic
Not at all

comments:

Do you consider that teaching within scientific methodology has/has had a fair amount of space during the education so far?
Yes, I think there is/has been adequate/enough space in the education
No, there is/has been too little space in the education
No, there is/has been too much space in the education

comments:

Do you consider that your scientific approach/knowledge needs to be strengthened?
No
Yes

(Filter: if no)
Why not:
My approach and knowledge/skills are already sufficient
I don't think a scientific approach is so important
Other reason:

What do you think would be needed to strengthen your scientific approach and -ability?

Other comments:

Your age (years)

What gender do you identify as?
Female
Male
Don’t want to disclose/state

At which university do you study?
Göteborgs Universitet
Karolinska Institutet
Linköpings Universitet
Luleå Tekniska Universitet
Lunds universitet
Mälardalens Högskola
Umeå universitet
Uppsala Universitet
